# Complete validation of plumbline distances as a screening tool for sagittal plane deformities

**DOI:** 10.1186/1748-7161-7-S1-O16

**Published:** 2012-01-27

**Authors:** S Negrini, S Donzelli, F Zaina, K Heitman, G Frattocchi, M Mangone

**Affiliations:** 1 ISICO (Italian Scientific Spine Institutes) Milan, Italy; 2 Diers International, GMBH, Germany; 3 Cattedra Medicina Fisica e Riabilitativa, Università La Sapienza, Roma, Italy

## Background

While for scoliosis screening Scoliometer has been widely validated, there is no validated screening instrument for sagittal plane deformities.

## Purpose

To validate a screening tool for sagittal plane deformities (plumbline distances - PD).

## Material and methods

Surface measurements (Formetric) of kyphosis/lordosis were considered the Gold Standard [1]. Correlations between Human PD (HPD), Formetric PD (FPD) and Gold Standard were searched in 129 school screening pupils (age 11.8±0.7): not correlated PD were eliminated. ROC-curve statistical technique was used to determine the best cut-off for remaining PDs.

Final FPD were verified in 7257 Formetric evaluations from the Diers database (3 age groups: 6-9y12m, 10-17y12m, 18-78). Final HPD were verified in 103 scoliosis/hyperkyphosis patients aged 14.3±2.2.

## Results

HPDs correlate with FPDs (0.49-0.57), C7+L3 with kyphosis (0.54-0.58), L3 with kyphosis and lordosis (0.42-0.56). To identify 60° kyphosis, a cut-off of 90 mm for C7+L3 demonstrated an overall accuracy range of 75-93%, high specificity (78-95%), variable sensitivity (25-83%). HPDs very well ruled out normals (negative predictive value –PV 93-99%), even if with high numbers of false positives (positive predictive value +PV 8-25%). Similarly, for 55° lordosis, a cut-off of 45 mm for L3 demonstrated a 75-94% overall accuracy, 70-94% specificity and 25-100% sensitivity, with –PV 93-100% and +PV 9-20%.

## Conclusions

In all groups evaluated results were similar. Below 90mm C7+L3 (45mm L3) almost all pupils are below 60° kyphosis (55° lordosis); in the remaining 20% a not-ionizing surface evaluation (Formetric) should be proposed to identify real deformities (1 out of 4 to 10).
